# Knee-Extension Training with a Single-Joint Hybrid Assistive Limb during the Early Postoperative Period after Total Knee Arthroplasty in a Patient with Osteoarthritis

**DOI:** 10.1155/2016/9610745

**Published:** 2016-09-28

**Authors:** Tomokazu Yoshioka, Hisashi Sugaya, Shigeki Kubota, Mio Onishi, Akihiro Kanamori, Yoshiyuki Sankai, Masashi Yamazaki

**Affiliations:** ^1^Division of Regenerative Medicine for Musculoskeletal System, Faculty of Medicine, University of Tsukuba, 1-1-1 Tennodai, Tsukuba, Ibaraki 305-8575, Japan; ^2^Department of Orthopedic Surgery, Faculty of Medicine, University of Tsukuba, 1-1-1 Tennodai, Tsukuba, Ibaraki 305-8575, Japan; ^3^Faculty of Systems and Information Engineering, University of Tsukuba, 1-1-1 Tennodai, Tsukuba, Ibaraki 305-8577, Japan

## Abstract

The knee range of motion is an important outcome of total knee arthroplasty (TKA). According to previous studies, the knee range of motion temporarily decreases for approximately 1 month after TKA due to postoperative pain and quadriceps dysfunction following surgical invasion into the knee extensor mechanism. We describe our experience with a knee-extension training program based on a single-joint hybrid assistive limb (HAL-SJ, Cyberdyne Inc., Tsukuba, Japan) during the acute recovery phase after TKA. HAL-SJ is a wearable robot suit that facilitates the voluntary control of knee joint motion. A 76-year-old man underwent HAL-SJ-based knee-extension training, which enabled him to perform knee function training during the acute phase after TKA without causing increased pain. Thus, he regained the ability to fully extend his knee postoperatively. HAL-SJ-based knee-extension training can be used as a novel post-TKA rehabilitation modality.

## 1. Introduction

The knee range of motion is an important outcome of total knee arthroplasty (TKA), a procedure commonly used to treat osteoarthritis of the knee [1]. According to previous studies, the knee range of motion decreases temporarily for approximately 1 month after TKA due to postoperative pain and quadriceps dysfunction following surgical invasion of the knee extensor mechanism. These previous studies have also indicated that this decrease in the knee range of motion correlates significantly with decreases in joint function and the patient's degree of satisfaction [2, 3]. Currently, no joint function exercises intended to maintain the range of passive knee extension obtained through surgery can be performed without pain, even when using active extension. Therefore, a new treatment strategy is needed to prevent the prolongation of extension lag after TKA.

**Figure 1:**
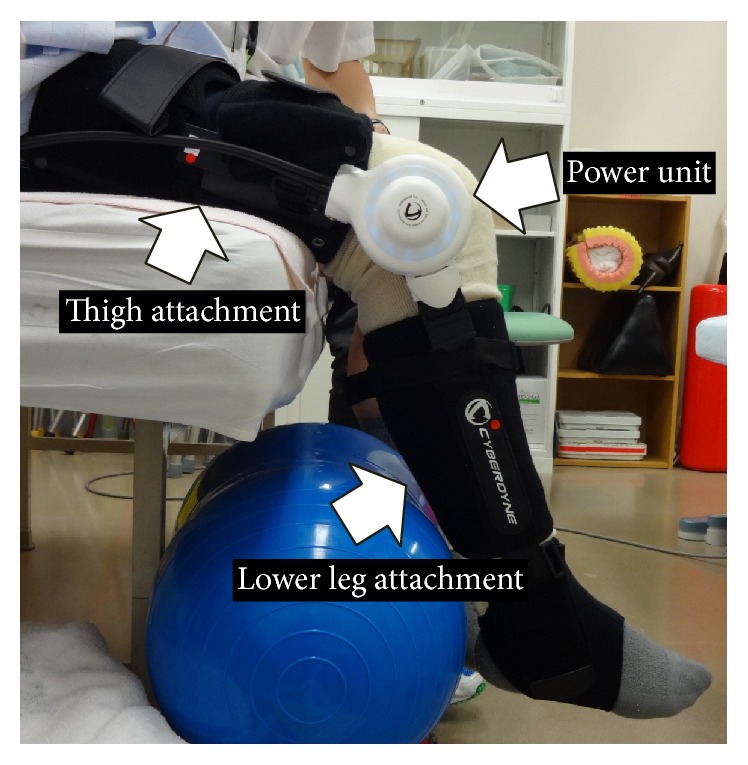
Lateral image of the single-joint hybrid assistive limb on the patient's right knee joint. Thigh and lower leg attachments are adjusted to the patient's body and connected by a power unit.

The single-joint hybrid assistive limb (HAL) (HAL-SJ, Cyberdyne Inc., Tsukuba, Japan) is a wearable robot suit that facilitates the voluntary control of knee joint motion (Figure 1). With this suit, signals from muscle action potentials are detected through electrodes on the surface of the skin and processed through a computer, after which the patient is provided with assisted joint motions. The power unit on the knee joint comprises angular sensors and actuators, and the control system comprises a cybernetic voluntary control (CVC) and cybernetic autonomous control (CAC) system [4]. The HAL has been reported to be effective in the functional recovery of various mobility disorders [5–8]. Although studies have reported successful outcomes for acute or chronic mobility disorders, there have been no reports on the use of HAL-SJ for degenerative joint diseases or related postoperative recovery to date. Accordingly, we describe our experience with a HAL-SJ-based knee-extension training program during the acute recovery phase after TKA.

## 2. Case Presentation

**Figure 2:**
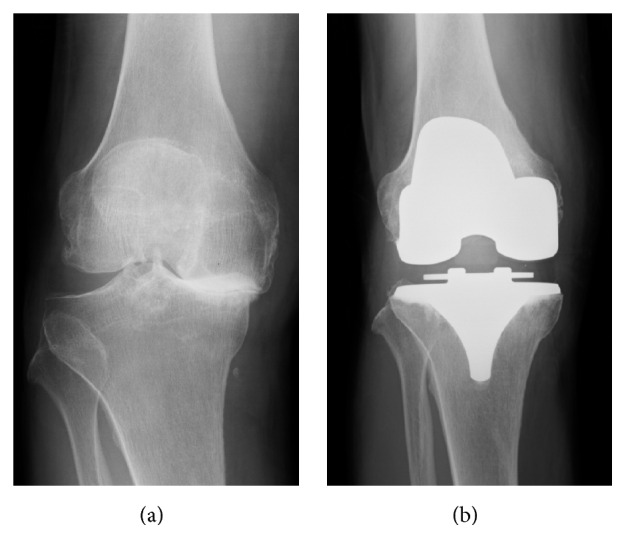
Preoperative (a) and postoperative (b) frontal radiographs of the knee.

**Figure 3:**
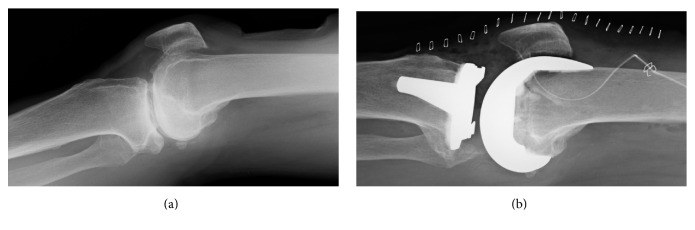
Lateral radiographs of the knee. (a) Preoperative passive knee extension without anesthesia. (b) Passive extension under postoperative anesthesia immediately postoperatively. Full knee extension was restricted preoperatively but it was possible immediately postoperatively.

A 76-year-old man underwent right TKA (Vanguard, Zimmer Biomet Inc., Warsaw, IN, USA) for grade 4 (Kellgren-Lawrence scale) osteoarthritis of the knee (Figures 2 and 3). The HAL treatment program was divided into the following five phases.

### 2.1. Preoperative Observation Phase (Day of Hospital Admission to the Day of Surgery)

The patient's thigh circumference and lower leg length were measured preoperatively, thus allowing us to adjust the HAL-SJ to the patient's size to ensure accurate training (Figure 1). We palpated the patient's quadriceps muscles (vastus medialis, rectus femoris, and vastus lateralis) and attached electrodes to each muscle to detect the bioelectric potentials of the long axes along the belly of each muscle. Then, we instructed the patient to perform knee-extension exercises and contract his quadriceps. We asked the patient to simulate the knee-extension training exercises, which were to be performed postoperatively, by performing 10 knee extensions with HAL-SJ assistance; the muscle that exhibited the highest bioelectric potential amplitude was used. The patient sat with his lower leg hanging down naturally, and we adjusted the height of the chair so his feet were not in contact with the floor (Figure 1).

### 2.2. Surgery Phase (Day of Surgery)

TKA was performed through a longitudinal incision with a medial parapatellar approach. We cemented the femoral and tibial components using the modified gap technique and a posterior stabilized-type device.

### 2.3. Postoperative Observation Phase (Postoperative Days 1–7)

**Figure 4:**
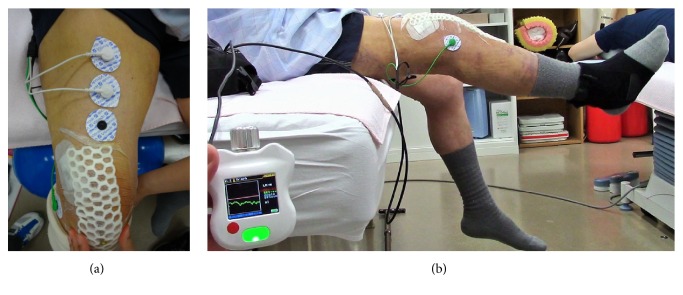
Bioelectric potential detection and simulation before single-joint hybrid assistive limb training. Electrodes were attached to the muscle belly of the quadriceps (a), and rectus femoris simulation (b) was performed. Electrodes were placed to avoid surgical wound.

On the first day after surgery, the patient was able to place full body weight on his leg; subsequently, he began rehabilitation (sitting, standing, and walking training; joint range of motion training; muscle strength maintenance; and muscle strengthening training) under the guidance of a physical therapist. Until discharge, he engaged in rehabilitation exercises for 20–40 min 5 days per week. Continuous passive motion (CPM) training began on the second postoperative day after the intra-articular drain was removed, and it was performed for 1 hour per day until discharge. On the seventh postoperative day, we attached electrodes to the quadriceps muscle again to detect the bioelectric potential along the long axis of the rectus femoris muscle belly (Figure 4(a)). Then, the patient was instructed to perform active knee-extension exercises to contract his quadriceps and thus simulate training with the HAL-SJ (Figure 4(b)).

### 2.4. HAL-SJ Therapy Phase (Postoperative Day 8 to Discharge)

**Figure 5:**
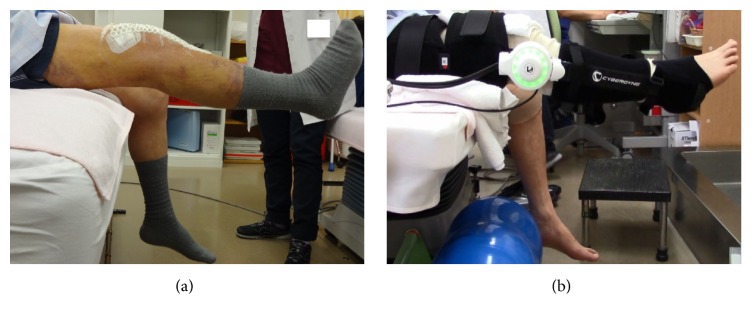
Knee-extension training on postoperative day 8. Active knee extension (a) did not result in full extension, whereas extension with single-joint hybrid assistive limb assistance resulted in full knee extension (b).

After 1 week of postoperative observation, we confirmed that his general condition had stabilized, and we decided to initiate HAL-SJ therapy. The CVC mode of the HAL-SJ, which was used in this study, can support a patient's voluntary motion according to the voluntary muscle activity and assistive torque provided to the knee joint [7]. This mode also allows the operator to adjust the degree of physical support to achieve patient comfort while gradually reducing support as training progresses. In addition to conventional rehabilitation (Figure 5(a)), the patient also performed HAL-SJ-assisted knee-extension exercises in a seated position at a frequency of 10 exercises/set for 5 sets twice weekly (HAL-SJ range of motion: 0–120°; Figure 5(b)). Training was performed 3 times (postoperative days 8, 10, and 17). The mean duration of a HAL-SJ training session was 26 min, which included the total time for which the HAL-SJ was worn and the duration of training (39, 22, and 17 min on postoperative days 8, 10, and 17, resp.).

### 2.5. Post-HAL-SJ Therapy Observation Phase (Discharge to 3 Months after the End of HAL-SJ Therapy)

There were no adverse effects related to HAL-SJ training. The patient was able to walk using a T cane, and he was discharged on postoperative day 21. Posttherapy assessments were conducted on an outpatient basis at 1 and 3 months after the end of the third HAL-SJ therapy session.

The following assessments were conducted: extension lag (maximum knee joint extension angle during passive exercise and that during active exercise), knee pain (visual analog scale, VAS), and isometric knee-extension muscle strength (IKEMS) before surgery, before and after HAL-SJ training, and at 1 and 3 months after training ended. The knee range of motion was measured using goniometry at accuracy of up to 1.0°, as goniometric measurements of range of motion have been reported to be more reliable than visual observation [9]. The measurement landmarks were the greater trochanter of the femur, proximal head of the fibula, and lateral malleolus. The maximal IKEMS of the operated leg was assessed while the patient was seated with 90° flexion in the hips and knees. Two measurements were taken using a *μ*Tas F-1 handheld dynamometer (Anima Corp., Tokyo, Japan) that was fixed to the chair. Each trial lasted for 3–5 s, with a 30-second rest period between trials. The higher of the two valid measurements was recorded. All measurements were performed by a single trained physical therapist to eliminate interobserver variability.

The extension lag, VAS, and IKEMS results are shown in Table 1. The extension lag was 15° preoperatively; this value decreased gradually over time to 1° at 3 months after therapy, indicating improvement. Comparisons before and after HAL-SJ therapy indicated that the 3 intervention sessions yielded respective improvements of 5°, 9°, and 5°. The VAS decreased from 55 mm before surgery to 17 mm at 3 months after the end of HAL-SJ therapy. Notably, training was not stopped because of increased knee pain from the HAL-SJ intervention. The maximum IKEMS value of 35.2 kgf was recorded before surgery. This value decreased markedly postoperatively and was measured as 18.3 kgf at 3 months after the end of the third HAL-SJ therapy session. Although this final value did not indicate recovery to the preoperative level, our comparison of IKEMS before and after HAL-SJ therapy indicated a slight improvement over the 3 intervention sessions (0.4, 0.0, and 1.8 kgf, resp.).

Clinical outcomes were assessed using the Japanese Orthopedic Association score [10]. The preoperative score of 55 points (pain, walking ability: 15 points; pain, ability to ascend/descend stairs: 5 points; flexion angle: 25 points; swelling: 10 points) improved to 90 points (pain, walking ability: 30 points; pain, ability to ascend/descend stairs: 20 points; flexion angle: 30 points; swelling: 10 points) at 3 months after the end of HAL-SJ therapy.

## 3. Discussion

The patient's clinical course described herein has yielded two important clinical findings. First, knee-extension training with HAL-SJ, performed as part of the acute phase of post-TKA rehabilitation, resulted in immediate improvements in extension lag. Second, knee-extension training with HAL-SJ could be performed without increased pain.

We will first address the immediate improvement in extension lag. According to a recent review, CPM with a knee-range-of-motion training device commonly used for acute post-TKA rehabilitation resulted in early improvements in the knee flexion range of motion, compared to not using CPM; however, neither the range of active nor that of passive knee extension improved with CPM [11]. Restricted post-TKA knee extension (decreased or poor extension range) has been significantly correlated with decreases in the Oxford Knee Score and clinical outcomes related to standing, as indicated by the Short Form-36 physical component score [2]. Therefore, maintenance of the improved knee-extension range obtained through surgery is extremely important to improving knee function. In the present study, HAL-SJ-based knee-extension training led to an immediate improvement in extension lag even though the quadriceps did not exhibit significant strengthening. This result suggests that this improvement resulted from the facilitation of the muscular and neural functions of the quadriceps by HAL-SJ, which allowed the knee to extend fully because of the presence of a bioelectric potential in the quadriceps and the degree of feedback strength.

As mentioned before, HAL-SJ-based knee-extension training, even during the acute postoperative stage, did not cause an increase in knee pain. Although isometric quadriceps training is performed during the acute post-TKA phase to address decreased or dysfunctional knee extension related to surgical invasion of the knee-extension mechanism, it is difficult for patients to sufficiently perform knee-extension training because of pain and swelling caused by the operation [2, 11, 12]. HAL-SJ-based knee-extension training, however, can be performed during the acute post-TKA phase without increased pain. We believe that this is due to the knee-assistive function of HAL-SJ.

Two case reports on postoperative interventional training using HAL have been published in the field of orthopedic surgery. Both reports described improvements in walking ability when HAL was used in patients with thoracic vertebra ossification of the posterior longitudinal ligament [8, 13]. In contrast, the present study is the first to report on the use of HAL-SJ knee-extension training during the acute phase following TKA for osteoarthritis of the knee.

In conclusion, HAL-SJ-based knee-extension training allows the performance of knee function training during the acute post-TKA phase without causing increased pain, thus maintaining the patient's surgically recovered ability to fully extend the knee. Although inability to fully extend the knee is a cause of reduced knee function and decreased satisfaction in patients after TKA, there is currently no effective modality for the recovery of knee-extension function. Therefore, HAL-SJ-based knee-extension training can be used as a novel post-TKA rehabilitation modality. Reduced medical costs can also be anticipated, as early recovery of knee function would reduce hospital stays and the nursing care burden consequent to improved patient independence. However, the mechanism underlying the immediate improvement in extension lag remains unknown; therefore, further study from a neurophysiological perspective is required.

## Figures and Tables

**Table 1:** Chronological changes in EL, VAS, and IKEMS.

	Preoperative	First HAL-SJ (^*∗∗∗*^POD 8)		Second HAL-SJ (POD 10)		Third HAL-SJ (POD 17)		At discharge (POD 21)	Following the end of the third HAL-SJ	
		^*∗*^IPO	^*∗∗*^IFO	IPO	IFO	IPO	IFO		1 month	3 months
EL (degrees)	15	10	5	12	3	10	5	4	4	1
VAS (mm)	55	28	4	20	20	32	46	40	18	17
IKEMS (kg)	35.2	8.7	9.1	5.6	5.6	10.7	12.5	16.9	16.9	18.3

EL: extension lag; VAS: visual analog scale; IKEMS: isometric knee-extension muscle strength; HAL-SJ: single-joint hybrid assistive limb.

^*∗*^IPO: immediately before the intervention.

^*∗∗*^IFO: immediately following the intervention.

^*∗∗∗*^POD: postoperative day.
